# Immune suppression in MTAP-deficient cancers via glutamate metabolism and CXCL10 downregulation

**DOI:** 10.3389/fimmu.2025.1634342

**Published:** 2025-10-30

**Authors:** Wen-Hsin Chang, Jun Zhang, Qi-Sheng Hong, Ching-Hsien Chen

**Affiliations:** ^1^ Division of Pulmonary, Critical Care and Sleep Medicine, Department of Internal Medicine, University of California, Davis, Davis, CA, United States; ^2^ Division of Nephrology, Department of Internal Medicine, University of California, Davis, Davis, CA, United States; ^3^ Comprehensive Cancer Center, University of California, Davis, Sacramento, CA, United States; ^4^ Graduate Institute of Medical Sciences, College of Medicine, Taipei Medical University, Taipei, Taiwan

**Keywords:** MTAP deficiency, immunosuppression, tumor microenvironment, CXCL10, glutamate metabolism

## Abstract

**Background:**

Immune checkpoint inhibitors (ICIs) have transformed cancer therapy; however, their efficacy remains limited in certain tumor subtypes, including those deficient in methylthioadenosine phosphorylase (MTAP). MTAP-deficient cancers are characterized by immunosuppressive tumor microenvironments (TMEs) and poor T cell infiltration, as suggested by large-scale transcriptomic analyses. Yet, the underlying mechanisms and therapeutic vulnerabilities remain poorly defined.

**Methods:**

We employed murine tumor models and transcriptomic profiling to investigate the immunosuppressive features of MTAP-deficient tumors. To identify actionable vulnerabilities, we conducted a high-throughput screen using the LOPAC1280 compound library. Functional assays were performed to evaluate the effects of candidate compounds on tumor growth and immune signaling.

**Results:**

MTAP-deficient tumors exhibited significantly reduced CD45+ immune cell infiltration and resistance to ICI therapy. Transcriptomic analyses revealed that MTAP-deficient cancer cells reprogram immune signaling pathways and suppress the expression of CXCL10, a key chemokine for T cell recruitment, thereby contributing to a non-inflamed, “cold” TME. High-throughput screening revealed an increased dependence on glutamate metabolism in MTAP-deficient cells. Several glutamate pathway inhibitors, including the clinically tested glutaminase inhibitor CB-839, selectively impaired their growth. Remarkably, CB-839 also restored CXCL10 expression, particularly under immune co-culture conditions, indicating a dual effect of direct cytotoxicity and immune activation.

**Conclusion:**

These findings uncover a novel link between glutamate metabolism and immune modulation in MTAP-deficient tumors. Our study provides mechanistic and preclinical support for targeting glutamate pathways to both suppress tumor growth and convert immune-cold tumors into more immunoresponsive states, offering a promising strategy to enhance ICI efficacy in this challenging cancer subtype.

## Introduction

Immune checkpoint inhibitors (ICIs) transformed the treatment landscape for multiple malignancies by offering superior clinical benefit and reduced toxicity compared to traditional chemotherapies (1, 2). U.S. Food and Drug Administration (FDA)-approved ICIs, such as the anti-PD-L1 antibody atezolizumab and the anti-PD-1 antibody nivolumab, have demonstrated therapeutic efficacy in cancers including non-small cell lung cancer (NSCLC) and renal cell carcinoma (RCC). Despite these advances, objective response rates (ORRs) remain modest, approximately 20-30% in most patients, due to intrinsic resistance mechanisms (3, 4). A major contributor to this limited efficacy is the presence of a non-inflamed or “cold” tumor microenvironment (TME), characterized by low immune infiltration and immunosuppressive signaling (5). Tumors with inadequate CD8^+^ cytotoxic T lymphocyte recruitment are less likely to respond to ICIs. Additionally, immune-related adverse events and the lack of reliable predictive biomarkers further limit their clinical utility. These challenges highlight the importance of understanding tumor-intrinsic mechanisms of immune resistance.

One emerging mechanism of immune evasion involves deletion of chromosome 9p21, which occurs in multiple cancer types and is associated with poor immune infiltration (6). Among the genes frequently lost in this region is methylthioadenosine phosphorylase (MTAP), an enzyme involved in the methionine and adenine salvage pathways. We previously demonstrated that MTAP functions as a metastasis suppressor (7, 8), and that MTAP-deficient cancer cells reprogram immune-related pathways and cytokine profiles, contributing to the establishment of an immunosuppressive “cold” TME (9). MTAP is an indispensable enzyme in catalyzing the breakdown of 5’-methylthioadenosine (MTA), a byproduct of polyamine synthesis (10). Loss of MTAP results in MTA accumulation, which can interfere with various signaling and epigenetic processes. In particular, MTA-mediated inhibition of methyltransferase-dependent pathways may impair chemokine/cytokine production, thereby contributing to immune suppression within the TME (11–13).

MTAP deficiency also profoundly alters tumor metabolism. Metabolomic analyses have shown that MTAP loss affects glycolytic flux and disrupts sulfur-containing amino acid and purine metabolism (14, 15). These metabolic changes create synthetic vulnerabilities that are being explored therapeutically. Current strategies include methionine restriction, inhibition of *de novo* purine synthesis, suppression of S-adenosylmethionine (SAM) production via MAT2A inhibitors, and inhibition of protein arginine methyltransferase 5 (PRMT5)-mediated arginine methylation (16–22). While preclinical studies support selective efficacy in MTAP-deficient tumors, clinical translation has been limited by toxicity and small patient cohorts. Moreover, whether these metabolic strategies can enhance antitumor immunity or synergize with ICIs remains largely unknown, underscoring the need to investigate the immunologic consequences of MTAP loss.

Given that MTAP deletion occurs in approximately 15% of human cancers (10), identifying novel, well-tolerated therapies that target both metabolic vulnerabilities and immunosuppressive features is a critical unmet need. In this study, we employed the Library of Pharmacologically Active Compounds (LOPAC1280), a high-throughput screening platform containing well-characterized small molecules, to identify agents capable of selectively suppressing tumor growth and modulating immune-relevant pathways in MTAP-deficient cancer cells. Our findings provide new insight into how tumor metabolism modulates immune resistance associated with MTAP loss, with potential implications for enhancing tumor immunogenicity and expanding the efficacy of immune-based therapies.

## Materials and methods

### Reagents

RPMI-1640 medium, fetal bovine serum, penicillin/streptomycin/Amphotericin B, and red blood cell (RBC) lysis buffer were purchased from Thermo Fisher Scientific (Waltham, MA, USA). Quantikine Human CXCL10/IP-10 ELISA Kit was purchased from R&D Systems (Minneapolis, MN, USA). CB-839 (telaglenastat) was purchased from Selleck Chemicals (Houston, TX, USA).

### Cell lines and cell culture

CL1–0 and 786–0 MTAP-intact (WT) and MTAP-knockout (KO) cells as well as CL1–5 and ACHN control (Mock) and MTAP-overexpressing (MTAP) cells were established and maintained at 37°C in a humidified atmosphere of 5% CO_2_ as previously described (7–9). To establish MTAP-knockout Lewis lung carcinoma (LLC) cells, we used lentiviruses generated by co-transfecting HEK293T cells with an MTAP sgRNA-containing lentiviral vector and a packaging DNA mix using Lipofectamine 2000. The lentiviral vector was constructed by synthesizing, annealing, and cloning the following oligonucleotides into the LentiCRISPRv2 expression vector: oligo 1 (5’- CACCGTCTCACCTTCACCGCCGTGC-3’) and oligo 2 (5’- AAACGCACGGCGGTGAAGGTGAGAC-3’). Cells were infected at three different Multiplicities of Infection (MOIs) in polybrene (8 µg/mL)-containing medium. Twenty-four hours after infection, the cells were treated with puromycin (final concentration 2 µg/mL) and puromycin-resistant clones were selected and sequenced to confirm the gene-editing results.

### *In vivo* animal experiments

All mouse experiments and procedures were approved and periodically reviewed by the Institutional Animal Care and Use Committee (IACUC) at UC Davis. Eight-week-old C57BL/6J mice were purchased from the Jackson Laboratory and housed four mice per cage and fed autoclaved food *ad libitum*. For tumorigenicity assay, 1×10^6^ cells were suspended in 100 μl PBS and implanted subcutaneously into the dorsal region of mice. Tumor growth was examined twice or thrice a week, and tumor volume was estimated by the formula *LW* ^2^/2, where *L* is the length and *W* is the width of the tumor. After 21 days, the mice were euthanized by CO_2_ inhalation at a displacement rate of 20% of the chamber volume per minute, and the tumor xenografts were removed, weighted, and photographed.

### Hematoxylin and eosin staining and immunohistochemistry

Formalin-fixed, paraffin-embedded (FFPE) tissue sections (4 µm) were deparaffinized, rehydrated, and stained using the Hematoxylin and Eosin Stain Kit (Vector Laboratories, Burlingame, CA, USA) according to the manufacturer’s protocol. FFPE sections were also used for immunohistochemical analysis of MTAP and CD45 expression. For immunohistochemistry, the protocol was adapted from the manufacturer’s paraffin immunohistochemistry guidelines (Cell Signaling Technology, Danvers, MA, USA). Tissue sections were deparaffinized in a xylene substitute, rehydrated through graded alcohol solutions, and subjected to antigen retrieval using 10 mM sodium citrate (pH 6.0) at a sub-boiling temperature. Endogenous peroxidase activity was blocked with 3% hydrogen peroxide, followed by serum blocking. Sections were incubated overnight at 4°C with anti-MTAP antibody (Novus Biologicals, Littleton, CO, USA) or anti-CD45 antibody (Cell Signaling Technology, Danvers, MA, USA). Immunostaining was detected using the VECTASTAIN ABC system (Vector Laboratories, Burlingame, CA, USA) following the manufacturer’s instructions. Stained sections were mounted and examined under a light microscope.

### Peripheral blood mononuclear cells isolation and co-culture

PBMCs were isolated from whole blood (STEMCELL Technologies, Cambridge, MA, USA) using a density gradient centrifugation method. For co-culture assays, cancer cells were mixed with isolated PBMCs at a ratio of 1:10 and co-cultured for 24 hours. After co-culture, cancer cells and PBMCs were separately harvested, washed with PBS, and processed for further assays.

### Real-time quantitative reverse transcription PCR

Total RNA was extracted from cancer cells using the TRIzol reagent (Invitrogen, Carlsbad, CA, USA). cDNAs were reversely transcribed using SuperScript III Reverse Transcriptase (Invitrogen). The primers used were as follows: CXCL9 forward primer 5’-CCAGTAGTGAGAAAGGGTCGC-3’ and reverse primer 5’-AGGGCTTGGGGCAAATTGTT-3’; CXCL10 forward primer 5’-CCAATTTTGTCCACGTGTTGAG-3’ and reverse primer 5’-GCTCCCCTCTGGTTTTAAGGA-3’; CXCL11 forward primer 5’-TTAAACAAACATGAGTGTGAAGGG-3’ and reverse primer 5’-CGTTGTCCTTTATTTTCTTTCAGG-3’; SLC38A2 forward primer 5’-AGCCAACAGCTCTTGTACCTGC-3’ and reverse primer 5’-GGAAGAACAGCAGGATGACAGAC-3’; SLC3A2 forward primer 5’-CCAGAAGGATGATGTCGCTCAG-3’ and reverse primer 5’-GAGTAAGGTCCAGAATGACACGG-3’; SLC7A5 forward primer 5’-GCCACAGAAAGCCTGAGCTTGA-3’ and reverse primer 5’-ATGGTGAAGCCGATGCCACACT-3’; SLC7A11 forward primer 5’-TCCTGCTTTGGCTCCATGAACG-3’ and reverse primer 5’-AGAGGAGTGTGCTTGCGGACAT-3’; TBP forward primer 5’-CACGAACCACGGCACTGATT-3’ and reverse primer 5’-TTTTCTTGCTGCCAGTCTGGAC-3’. The housekeeping gene TBP was utilized as the reference gene in quantitative real-time RT-PCR assay. Quantitative real-time RT-PCR was conducted using the SYBR Green system and performed according to the manufacturer’s instructions of the ViiA 7 Real-Time PCR System (Applied Biosystems, Thermo Fisher Scientific, Waltham, MA, USA). The relative expression level of the target gene compared with that of the TBP was defined as –ΔCT = – [CT_target gene_ – CT_TBP_]. The target gene/TBP mRNA ratio was calculated as 2^-ΔCT^ x K, where K is a constant.

### Promoter-luciferase reporter assay

The constructs containing 1000 bp (1K) or 2000 bp (2K) upstream of the CXCL10 transcription start site were cloned into the pGL3-Basic luciferase reporter vector (Promega Corporation, Madison, WI, USA). The resulting constructs were verified by DNA sequencing. CL1–0 cells were co-transfected with the CXCL10 promoter-luciferase constructs and the phRL-TK vector, which served as an internal control to normalize for transfection efficiency. The pGL3-Basic empty vector was used as a negative control. Luciferase activity was measured using the Dual-Luciferase Reporter Assay Kit (Promega), following the manufacturer’s instructions. Firefly luciferase activity was normalized to Renilla luciferase activity to calculate relative promoter activity for each condition.

### Immunoblotting

The cells were collected and prepared as whole-cell lysates with lysis buffer (50 mM Tris-HCl [pH 7.4], 1% Triton X-100, 10% glycerol, 150 mM NaCl, 1 mM EDTA, 20 μg/mL leupeptin, 1 mM PMSF, and 20 μg/mL aprotinin). Total proteins were separated via SDS-PAGE and transferred to polyvinylidene fluoride (PVDF) membranes, followed by immunoblotting with anti-phospho-STAT1, anti-STAT1, anti-phospho-p65, anti-p65, anti-β-actin (Cell Signaling Technology, Danvers, MA, USA), anti-MTAP (Novus Biologicals, Littleton, CO, USA) antibodies and chemiluminescence detection. The protein expression levels were quantified by ImageJ software (National Institutes of Health, Bethesda, MD, USA).

### LOPAC 1280 compound library screening and drug treatment

The LOPAC 1280 library (Sigma-Aldrich, Cat# LO1280) was used to identify selective inhibitors for MTAP-deficient cancer cells. CL1–0 MTAP-WT and MTAP-KO cells were seeded in 96-well plates and treated the following day with either vehicle (DMSO) or individual compounds at 2 or 20 μM for 72 hours. Cell viability was then assessed using MTT assays. Compounds exhibiting dose-dependent cytotoxicity and a viability difference greater than 40% between MTAP-WT and MTAP-KO cells were selected for further analysis.

### Statistical analysis

Data from at least three independent experiments are presented as the mean ± standard deviation (SD). Quantitative variables were analyzed using an unpaired two-tailed Student’s t-test. All analyses were performed using GraphPad Prism software (version 10.4.1, Boston, MA, USA). Statistical tests were two-sided, and *p*-values < 0.05 were considered statistically significant.

## Results

### MTAP deficiency promotes tumor growth and reduces immune cell infiltration *in vivo*

**Figure 1:**
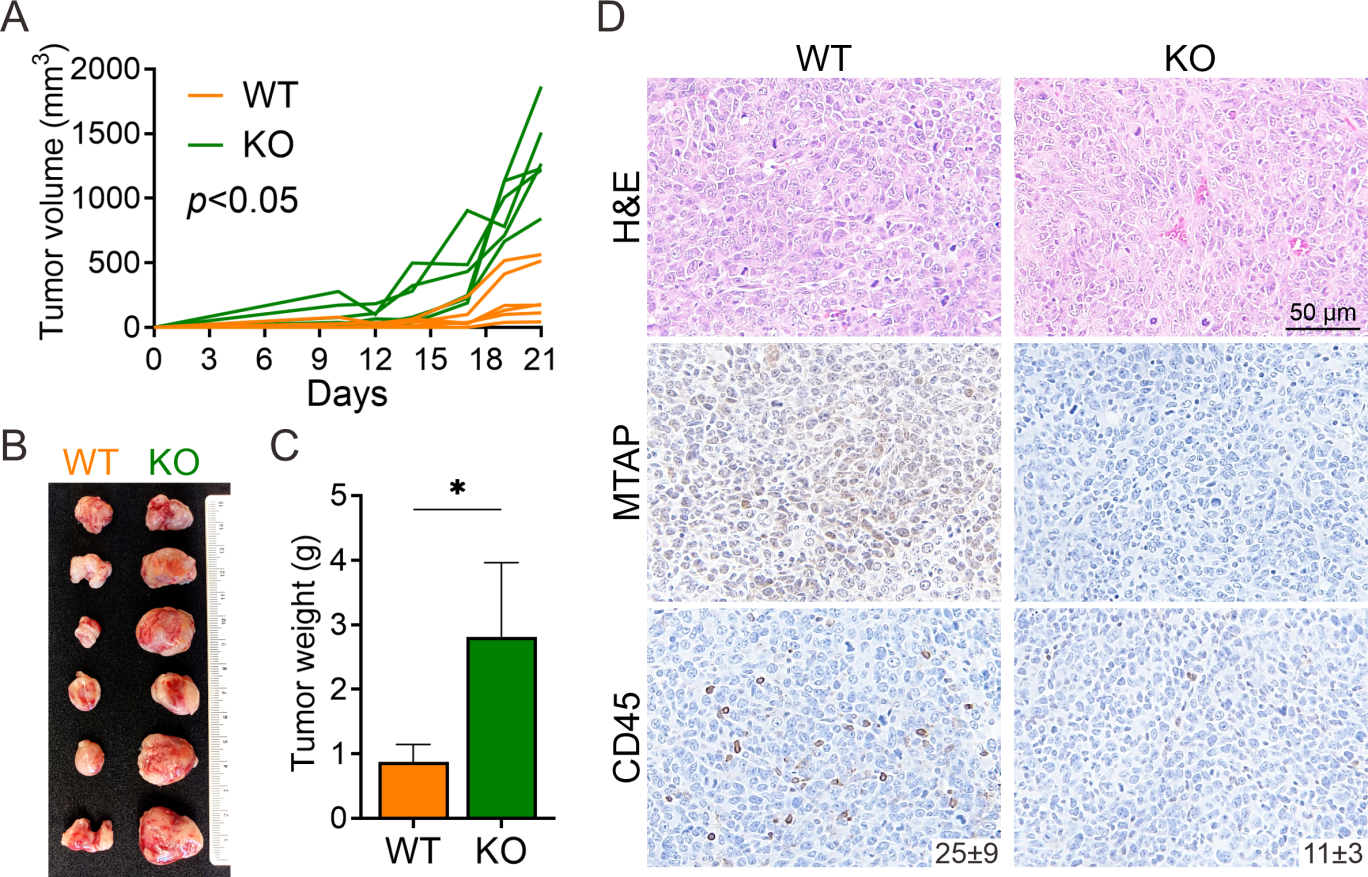
Murine model of MTAP-deficient cancer. **(A)** Tumor growth of subcutaneously injected MTAP-WT and MTAP-KO LLC murine lung cancer cells in C57BL/6J mice was measured every 2–3 days and analyzed at Day 21 (six mice per group). **(B)** A representative photo of primary tumors is shown. Each row of paired tumors was derived from the same mouse. **(C)** Tumor weight was measured 21 days post-injection (*p < 0.05). **(D)** Representative images of H&E staining and MTAP and CD45 IHC staining in tumor sections. Numbers indicate the count of CD45+ cells per field.

To assess the role of MTAP in regulating antitumor immunity, we employed an immunocompetent murine model using Lewis lung carcinoma (LLC) cells with or without MTAP expression. MTAP-intact (wild-type, WT) and MTAP-knockout (KO) cells were subcutaneously injected into the contralateral flanks of syngeneic C57BL/6J mice. By day 21, tumors derived from MTAP-KO cells exhibited significantly accelerated growth and greater tumor volume compared to their WT counterparts (**Figure 1A**). Consistently, excised MTAP-KO tumors were markedly larger and heavier than MTAP-WT tumors across all animals (**Figures 1B,C**). Histological evaluation revealed notable differences in tumor architecture. Hematoxylin and eosin (H&E) staining indicated increased vascularization and stromal remodeling in MTAP-KO tumors relative to WT (**Figure 1D**, top), top. Immunohistochemical analysis confirmed strong MTAP expression in WT tumors and its absence in KO tumors (**Figure 1D, middle**). Notably, CD45^+^ immune cell infiltration was significantly reduced in MTAP-deficient tumors, suggesting impaired recruitment of leukocytes (**Figure 1D, bottom**). These findings suggest that MTAP loss enhances tumor progression and is associated with reduced immune cell infiltration, highlighting the importance of MTAP in antitumor immune responses.

### Transcriptomic analysis reveals impaired immune signaling and reduced CXCL10 expression in MTAP-deficient cancer cells

**Figure 2:**
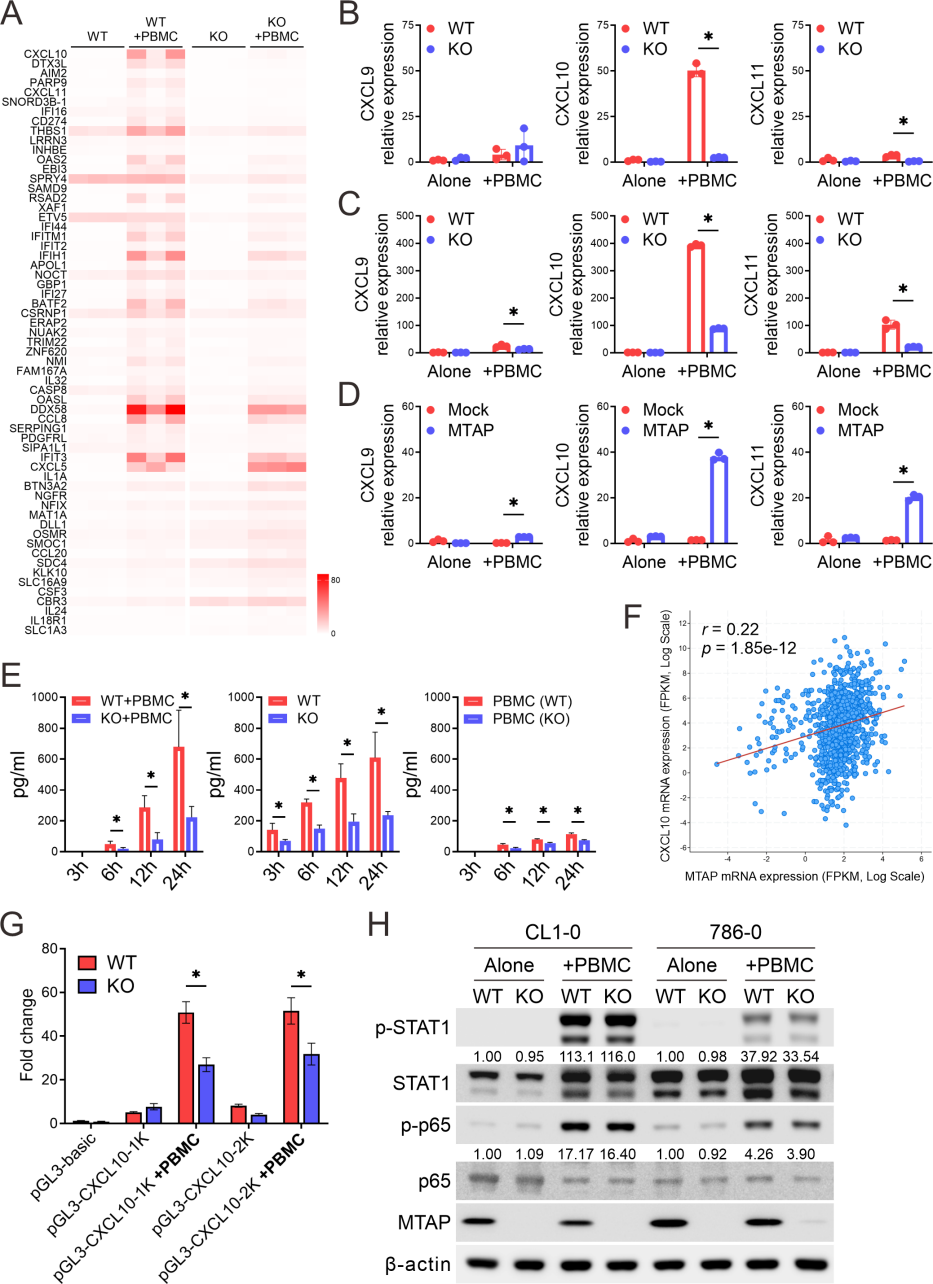
Dysregulation of CXCL10 in MTAP-deficient cancer. **(A)** Heatmap of FPKM values for differentially expressed genes in CL1–0 WT/KO cancer cells co-cultured with or without PBMCs, shown across three biological replicates. **(B-D)** RT-qPCR analysis of CXCL9, CXCL10, and CXCL11 mRNA expression in MTAP-expressing and MTAP-deficient CL1-0 **(B)**, 786-0 **(C)**, and CL1-5 **(D)** cancer cells co-cultured with PBMCs (*p < 0.05). **(E)** ELISA analysis of CXCL10 levels in conditioned media from CL1–0 WT/KO cancer cells co-cultured with PBMCs (left), isolated cancer cells (middle), and isolated PBMCs (right) at 3, 6, 12, and 24 hours. PBMCs were isolated from the peripheral blood of three individual donors. **(F)** Correlation between MTAP and CXCL10 expression in 991 patient samples from the TCGA pan-cancer dataset. *r* indicates the Pearson correlation coefficient. **(G)** Luciferase reporter assays of CXCL10 promoter activity in MTAP-WT and MTAP-KO CL1–0 cancer cells with or without PBMC co-culture. “1K” and “2K” indicate constructs containing the proximal 1-kb and 2-kb regions of the CXCL10 promoter, respectively. **(H)** Western blot analysis of phospho-STAT1 and phospho-p65 in MTAP-WT and -KO cancer cells following PBMC co-culture. Numbers indicate the quantification relative to β-actin from three independent Western blots.

To elucidate the molecular mechanisms by which MTAP regulates antitumor immune responses, we performed bulk RNA sequencing to analyze transcriptomic alterations in MTAP-WT (WT) and MTAP-KO (KO) cancer cells co-cultured with peripheral blood mononuclear cells (PBMCs) (WT+PBMC and KO+PBMC). We focused on genes with a fragments per kilobase of transcript per million mapped reads (FPKM) value ≥1 and identified those with at least a two-fold change in expression when comparing WT vs. WT+PBMC, KO vs. KO+PBMC, and WT+PBMC vs. KO+PBMC. This analysis yielded 61 differentially expressed genes, ranked by fold change between WT+PBMC and KO+PBMC (**Figure 2A**). Of these, 43 genes were upregulated in the WT+PBMC condition, while only 18 genes were upregulated in KO+PBMC cells, supporting our *in vivo* findings that MTAP deficiency suppresses immune activation. Notably, CXCL10 (IP-10, interferon gamma-induced protein 10), a chemokine critical for T cell recruitment and a positive prognostic marker for response to immune checkpoint blockade (23), was significantly downregulated in KO+PBMC cells. Additionally, several interferon-inducible (IFI) genes were also suppressed in MTAP-deficient cells, suggesting impaired interferon signaling in the absence of MTAP.

Since CXCL9, CXCL10, and CXCL11 are ligands for chemokine receptor CXCR3, and play a crucial role in leukocyte migration and activation (24), we further assessed their expression using RT-qPCR analysis. Baseline expression levels of CXCL9, CXCL10, and CXCL11 were relatively low and comparable between MTAP-WT and MTAP-KO CL1–0 and 786–0 cancer cells, as well as between CL1–5 Mock and MTAP-expressing cells (**Figures 2B–D**). However, upon co-culture with PBMCs, expression of these chemokines was robustly induced in MTAP-expressing cells but significantly blunted in MTAP-KO and Mock cells. Among the three, CXCL10 exhibited the most pronounced differential induction, further underscoring its central role in MTAP-mediated immune responses. To determine the source of CXCL10 production, we performed ELISA assays on culture supernatants and confirmed that CXCL10 secretion was significantly reduced in MTAP-KO cells, indicating that cancer cells, not PBMCs, were the primary contributors to CXCL10 levels (**Figure 2E**). Further analysis using the TCGA pan-cancer dataset (25) consolidated the positive correlation between MTAP and CXCL10 expression (**Figure 2F**). To explore upstream regulatory mechanisms, we conducted luciferase reporter assays using cloned CXCL10 promoter constructs. MTAP-KO cancer cells displayed significantly lower luciferase activity, and deletion mapping localized the responsible regulatory elements to the proximal 1-kb region of the CXCL10 promoter (**Figure 2G**). We further investigated potential upstream regulators of CXCL10 by examining STAT1 and NF-κB p65, two key transcription factors known to drive CXCL10 expression. Phosphorylation of both factors following PBMC co-culture was minimally affected by MTAP status, suggesting that CXCL10 suppression is not primarily mediated through impaired STAT1 or NF-κB signaling (**Figure 2H**). Collectively, these findings demonstrate that MTAP deficiency suppresses CXCL10 transcription and secretion in response to immune cell contact, thereby limiting chemokine-mediated immune cell recruitment. This suggests that MTAP plays a critical role in shaping tumor immunogenicity through regulation of interferon-driven chemokine expression.

### High-throughput screening identifies glutamate signaling as a vulnerability in MTAP-deficient cancer cells

**Figure 3:**
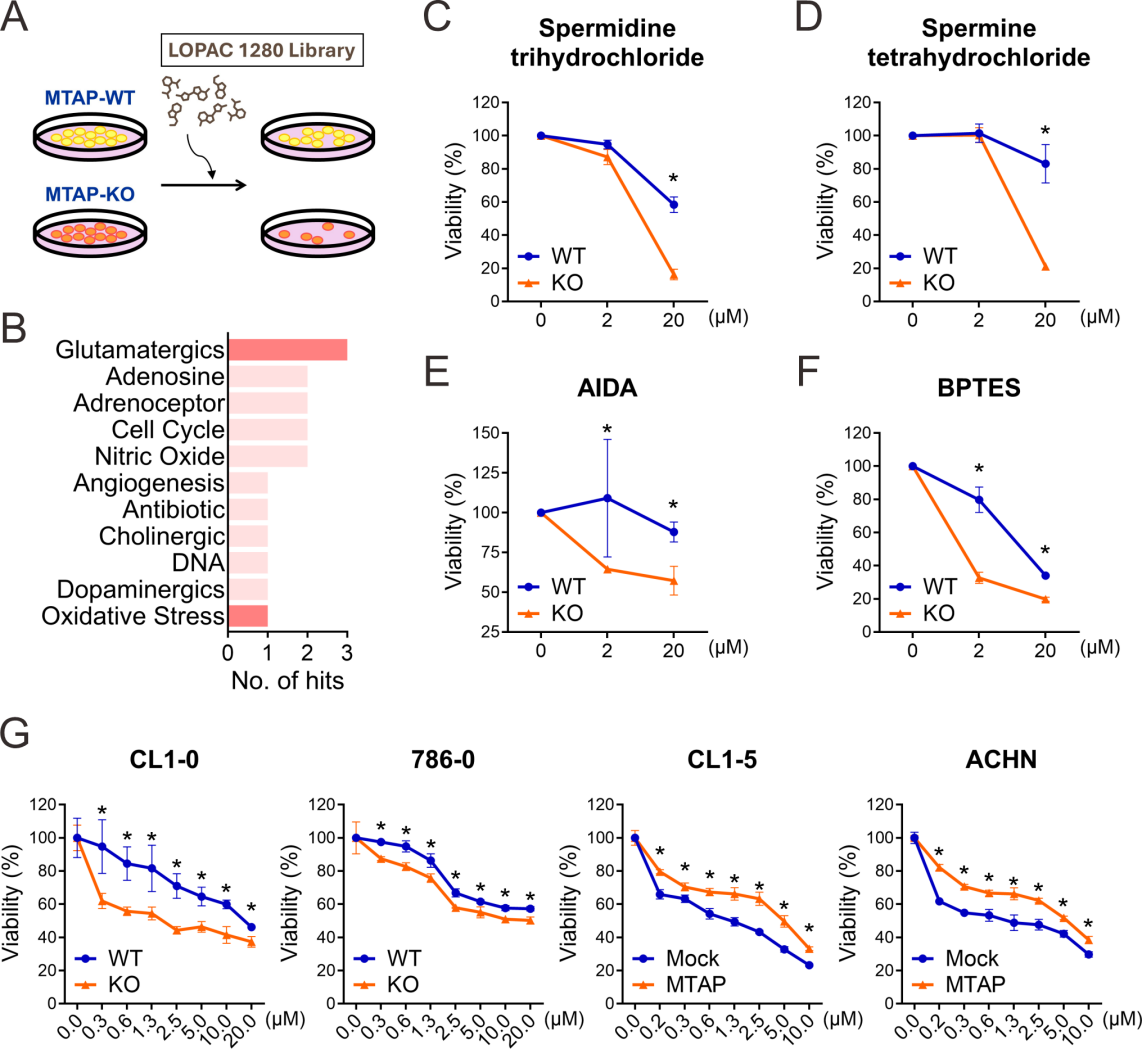
Screening of novel inhibitors targeting MTAP-deficient cancer. **(A)** Schematic overview of the drug screening strategy for identifying compounds selectively targeting MTAP-KO cells. **(B)** Classification of compounds from the LOPAC 1280 library based on selectivity for MTAP-KO cells. Red bars indicate glutamate-related pathways. **(C-F)** MTT assay results for spermidine trihydrochloride, spermine tetrahydrochloride, and AIDA (glutamatergic pathway) and BPTES (oxidative stress pathway) in MTAP-WT and MTAP-KO cells (*p < 0.05). **(G)** CB-839 treatment response in MTAP-expressing and MTAP-deficient cancer cells was assessed by MTT assays (*p < 0.05).

Having uncovered the mechanistic role of MTAP deficiency in promoting immune evasion, we next sought to identify therapeutic strategies that selectively target MTAP-deficient cancer cells while potentially enhancing antitumor immune responses. To this end, we utilized the LOPAC1280, a well-characterized high-throughput screening library composed of pharmacologically active small molecules (**Figure 3A**). CL1–0 MTAP-WT and MTAP-KO cells were screened across multiple dosages to evaluate compound specificity and differential sensitivity. Initial screening using MTT assays identified 53 candidate compounds with selective cytotoxicity against MTAP-KO cancer cells. Remarkably, four of these compounds were involved in glutamate-related signaling pathways (**Figure 3B**): spermidine trihydrochloride and spermine tetrahydrochloride are modulators of NMDA-type glutamate receptors (**Figures 3C,D**); AIDA acts as an antagonist of metabotropic glutamate receptors (**Figure 3E**); and BPTES inhibits glutaminase, a key enzyme in glutamine-to-glutamate conversion (**Figure 3F**). These findings suggest that MTAP-deficient cancer cells are particularly susceptible to disruption of glutamate signaling. To further explore this metabolic vulnerability, we tested CB-839, a clinically advanced analog of BPTES with improved solubility and potent antiproliferative activity (26). CB-839 was evaluated across four matched pairs of MTAP-expressing and MTAP-deficient cancer cell lines. Strikingly, CB-839 exhibited strong selective efficacy in MTAP-deficient cells at concentrations as low as 0.3 μM (**Figure 3G**). Together, these findings demonstrate that MTAP-deficient cancer cells display a critical dependence on glutamate metabolism. Targeting glutamate signaling may therefore represent a promising therapeutic strategy in MTAP-deleted malignancies.

### Glutaminase inhibition preferentially enhances CXCL10 expression in MTAP-deficient cancer cells

**Figure 4:**
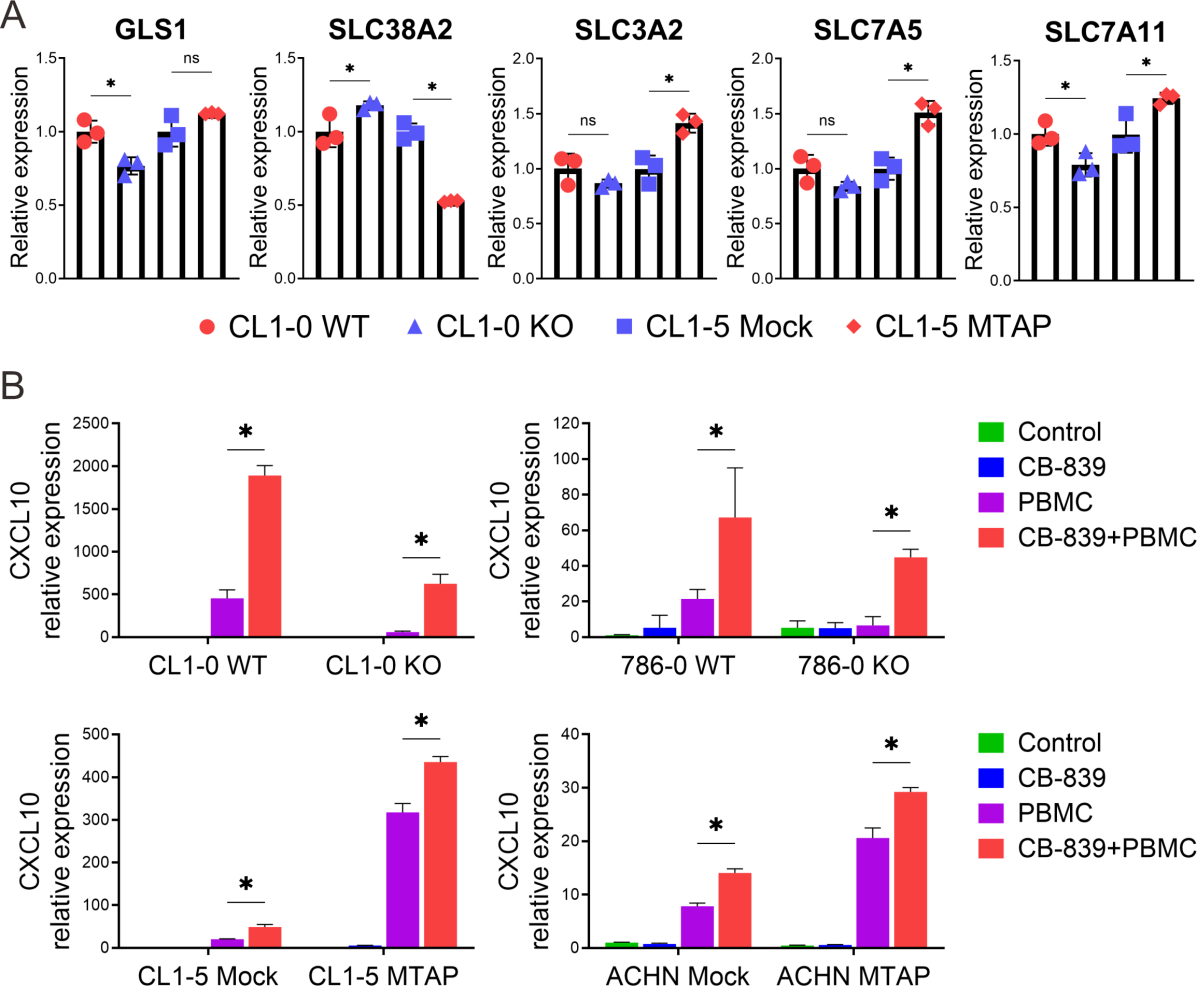
Alterations in glutamine transport and the effect of CB-839 on CXCL10 expression in MTAP-deficient cancer. **(A)** Expression analysis of glutaminase 1 (GLS1) and glutamine transporter genes SLC38A2, SLC3A2, SLC7A5, and SLC7A11 in MTAP-expressing and MTAP-deficient cancer cells by RT-qPCR assays. Expression levels were normalized to CL1–0 WT or CL1–5 Mock (*p < 0.05, ns: not significant). **(B)** RT-qPCR analysis of CXCL10 mRNA levels in MTAP-expressing and MTAP-deficient cancer cells under different conditions: treatment with/without 5 µM CB-839 for 24 hours and co-culture with/without PBMCs for 24 hours (*p < 0.05). Expression levels were normalized to the respective control groups: CL1–0 WT, 786–0 WT, CL1–5 Mock, and ACHN Mock.

To further understand the regulatory mechanisms underlying glutamate dependency in MTAP-deficient cancer cells, we examined the expression of glutaminase 1 (GLS1), the target of CB-839, and key glutamine/glutamate transporters. RT-qPCR analysis revealed a slight decrease in GLS1 expression in MTAP-deficient cells, despite their increased sensitivity to CB-839 treatment. Interestingly, MTAP-deficient cells showed a modest upregulation of the glutamine transporter SLC38A2, while the glutamine antiporter complex SLC3A2-SLC7A5 and the glutamate-cystine antiporter SLC7A11 were moderately downregulated (**Figure 4A**). These changes suggest a metabolic shift favoring increased glutamine uptake and reduced glutamate export, potentially reinforcing intracellular glutamate dependence in MTAP-deficient cells. Given the selective vulnerability of MTAP-deficient cancer cells to CB-839 treatment and the immunoregulatory role of CXCL10, we next evaluated whether glutaminase inhibition could enhance CXCL10 expression. As shown in **Figure 4B**, basal level of CXCL10 was minimal in cancer cells cultured alone (green bar). Co-culture with PBMCs induced CXCL10 expression in both MTAP-expressing and MTAP-deficient cells, but the magnitude of induction was significantly higher in MTAP-expressing cells (purple bar). While CB-839 treatment had little effect on CXCL10 expression (blue bar), pre-treatment with CB-839 followed by PBMC co-culture led to a marked increase in CXCL10, particularly in MTAP-deficient cells (red bar). These findings suggest that CB-839 effectively enhances CXCL10 expression in an immune-competent context, with preferential effects in MTAP-deficient tumors. This finding supports the potential of glutaminase inhibition not only to impair tumor metabolism but also to augment chemokine-driven immune responses, offering a dual therapeutic strategy for MTAP-deficient malignancies.

## Discussion

In this study, we demonstrated that MTAP plays a dual role in cancer by regulating both tumor progression and immune responses. MTAP-deficient cancer cells showed impaired induction of key chemokines, CXCL9, CXCL10, and CXCL11, particularly in response to immune cell interaction, thereby contributing to the development of an immunosuppressive “cold” tumor microenvironment. Through a targeted high-throughput screen using the LOPAC1280 compound library, we identified pharmacological agents capable of both suppressing malignant phenotypes and partially restoring immune-related gene expression in MTAP-deficient cells. These findings suggest new therapeutic avenues for overcoming the limitations of current MTAP-targeted strategies and enhancing antitumor immunity.

Previous large-scale functional screens identified PRMT5 as a synthetic lethal target in MTAP-deleted cancer cells (17–19). This led to the development of therapeutic strategies targeting the methionine-MAT2A-SAM-PRMT5-MTA axis (20). Subsequent efforts focused on MAT2A inhibition and more recently, MTA-cooperative PRMT5 inhibitors, which selectively exploit elevated MTA levels in MTAP-deficient tumors to achieve higher specificity while sparing MTAP-proficient cells (27–29). Several of these agents are currently undergoing evaluation in phase 1/2 clinical trials. While early data appear encouraging, concerns remain about toxicity to normal tissues and the long-term effects on the tumor immune landscape. Additionally, their potential to synergize with ICIs is yet to be explored in clinical settings.

In contrast, we focused on CB-839, a clinically advanced GLS inhibitor with a favorable safety profile and ongoing trials in various malignancies, including triple-negative breast cancer, non-small cell lung cancer, and mesothelioma (30), and metastatic renal cell carcinoma (31). Although clinical responses to CB-839 have been variable across tumor types (32, 33), our findings suggest that MTAP-deficient tumors represent a distinct subset with heightened vulnerability to GLS inhibition. CB-839 targets GLS, a key enzyme in glutamine metabolism that fuels glutamate and α-ketoglutarate production essential for tumor bioenergetics and redox balance (34). Importantly, CB-839 has been shown to improve ICI responses in murine melanoma models by remodeling the TME and enhancing effector T cell infiltration (35). In our study, CB-839 alone did not induce CXCL10 in cancer cells but selectively impaired the growth of MTAP-deficient cancer cells and restored CXCL10 expression under immune co-culture, suggesting a dual mechanism involving both tumor cell-intrinsic cytotoxicity and immune cell-mediated feedback. Further evaluation of CB-839 in combination with immunotherapy will be important to define the translational potential of integrating glutaminase inhibition with immune checkpoint blockade in MTAP-deficient tumors.

Altered tumor metabolism plays a central role in shaping immune responses by affecting nutrient competition, cytokine signaling, and immune cell function. Studies have shown that amino acids such as arginine, asparagine, aspartate, serine, and glutamine are often regionally depleted within tumor cores compared to the periphery (36). Among them, glutamine is particularly critical for mitochondrial metabolism, glutathione synthesis, and non-essential amino acid production (37). As a result, targeting glutamine metabolism has emerged as a promising therapeutic strategy. Approaches such as suppressing glutamine uptake via the ASCT2/SLC1A5 transporter (38) or inhibiting GLS activity (26, 39) have shown efficacy in preclinical models. Notably, ASCT2 inhibition with V9302 synergized with anti-PD-L1 therapy through an IFN-γ-driven mechanism (40), emphasizing the immunomodulatory potential of targeting glutamine metabolism. Our findings further support this concept: GLS inhibition via CB-839 not only selectively impaired MTAP-deficient tumor growth but also restored CXCL10 expression, particularly in immune co-culture. Given that CXCL10 is critical for T cell recruitment, these findings suggest that glutaminase inhibition may help reverse immune suppression in MTAP-deficient tumors and sensitize them to immunotherapy.

CXCL10 has emerged as a favorable prognostic biomarker for immunotherapy responsiveness across multiple cancer types (23, 41–43). Given its critical role in recruiting T cells, strategies to enhance CXCL10 expression in the TME are actively being explored as a means to improve immune infiltration and response to checkpoint blockade. At the transcriptional level, CXCL10 is regulated by STAT1 and NF-κB p65, which bind to IFN-stimulated and NF-κB response elements following IFN-γ or IL-1β signaling (44). However, our data indicate that CXCL10 suppression in MTAP-deficient cells may not be regulated through the two transcription factors. The cGAS-STING-TBK1-IRF3 pathway also induces CXCL10 expression as part of a type I interferon response to cytosolic DNA (45). Notably, many of these immune pathways can be modulated by protein arginine methyltransferases (PRMTs) in an MTA-sensitive manner (46–48), suggesting a mechanistic link between MTAP loss, MTA accumulation, and impaired chemokine signaling. Several recent strategies have been shown to boost CXCL10 levels, including combination regimens such as PEM/CDDP with MEK1/2 inhibitors, which enhance CD8+ T cell infiltration and synergize with anti-PD-L1 therapy (49). Moreover, CXCL10-engineered dendritic cell vaccines have shown promise in enhancing T cell recruitment and overcoming ICI resistance (50). These approaches highlight CXCL10 as a pivotal mediator of TME remodeling. Our data show that glutaminase inhibition can restore CXCL10 expression in MTAP-deficient cells, offering a promising strategy to “warm up” immunologically silent tumors.

Future mechanistic studies are warranted to define how MTAP deficiency alters tumor glutamate metabolism to suppress CXCL10 expression, and whether CB-839-mediated upregulation of CXCL10 is sufficient to restore T cell infiltration and activation in MTAP-deficient tumors. It will also be important to delineate the crosstalk between tumor cells and diverse immune cell populations within the TME, particularly in the context of metabolic modulation. In parallel, further investigation into potential resistance mechanisms, such as compensatory metabolic pathways or altered nutrient uptake, as well as the safety considerations identified in early-phase clinical trials, will be critical for advancing glutaminase inhibitors toward therapeutic application in MTAP-deficient cancers. In conclusion, this study reveals how MTAP loss contributes to immune evasion and identifies glutamate metabolism as a therapeutically actionable vulnerability. Our findings support further evaluation of glutaminase inhibition, particularly CB-839, as a means to restore antitumor immunity in MTAP-deficient cancers and to enhance the efficacy of immune checkpoint blockade in tumors that are otherwise resistant.

## Data Availability

The datasets presented in this study can be found in online repositories. The names of the repository/repositories and accession number(s) can be found below: https://www.ncbi.nlm.nih.gov/, PRJNA720919.

## References

[1] Sordo-Bahamonde C Lorenzo-Herrero S Gonzalez-Rodriguez AP Martinez-Perez A Rodrigo JP Garcia-Pedrero JM. Chemo-immunotherapy: A new trend in cancer treatment. Cancers (Basel). (2023) 15:2912. doi: 10.3390/cancers15112912, PMID: 37296876 PMC10252089

[2] Wei J Li W Zhang P Guo F Liu M. Current trends in sensitizing immune checkpoint inhibitors for cancer treatment. Mol Cancer. (2024) 23:279. doi: 10.1186/s12943-024-02179-5, PMID: 39725966 PMC11670468

[3] Kiyotani K Toyoshima Y Nakamura Y. Personalized immunotherapy in cancer precision medicine. Cancer Biol Med. (2021) 18:955–65. doi: 10.20892/j.issn.2095-3941.2021.0032, PMID: 34369137 PMC8610159

[4] Alsaafeen BH Ali BR Elkord E. Resistance mechanisms to immune checkpoint inhibitors: updated insights. Mol Cancer. (2025) 24:20. doi: 10.1186/s12943-024-02212-7, PMID: 39815294 PMC11734352

[5] Khosravi GR Mostafavi S Bastan S Ebrahimi N Gharibvand RS Eskandari N. Immunologic tumor microenvironment modulators for turning cold tumors hot. Cancer Commun (Lond). (2024) 44:521–53. doi: 10.1002/cac2.12539, PMID: 38551889 PMC11110955

[6] Han G Yang G Hao D Lu Y Thein K Simpson BS. 9p21 loss confers a cold tumor immune microenvironment and primary resistance to immune checkpoint therapy. Nat Commun. (2021) 12:5606. doi: 10.1038/s41467-021-25894-9, PMID: 34556668 PMC8460828

[7] Xu J Chang WH Fong LWR Weiss RH Yu SL Chen CH. Targeting the insulin-like growth factor-1 receptor in MTAP-deficient renal cell carcinoma. Signal Transduct Target Ther. (2019) 4:2. doi: 10.1038/s41392-019-0035-z, PMID: 30701095 PMC6345872

[8] Chang WH Chen YJ Hsiao YJ Chiang CC Wang CY Chang YL. Reduced symmetric dimethylation stabilizes vimentin and promotes metastasis in MTAP-deficient lung cancer. EMBO Rep. (2022) 23:e54265. doi: 10.15252/embr.202154265, PMID: 35766227 PMC9346486

[9] Chang WH Hsu SW Zhang J Li JM Yang DD Chu CW. MTAP deficiency contributes to immune landscape remodelling and tumour evasion. Immunology. (2023) 168:331–45. doi: 10.1111/imm.13587, PMID: 36183155 PMC9840685

[10] Bray C Balcells C McNeish IA Keun HC. The potential and challenges of targeting MTAP-negative cancers beyond synthetic lethality. Front Oncol. (2023) 13:1264785. doi: 10.3389/fonc.2023.1264785, PMID: 37795443 PMC10546069

[11] Dai W Zhang J Li S He F Liu Q Gong J. Protein arginine methylation: an emerging modification in cancer immunity and immunotherapy. Front Immunol. (2022) 13:865964. doi: 10.3389/fimmu.2022.865964, PMID: 35493527 PMC9046588

[12] Wang Z Li R Hou N Zhang J Wang T Fan P. PRMT5 reduces immunotherapy efficacy in triple-negative breast cancer by methylating KEAP1 and inhibiting ferroptosis. J Immunother Cancer. (2023) 11:e006890. doi: 10.1136/jitc-2023-006890, PMID: 37380368 PMC10410861

[13] Gao Y Feng C Ma J Yan Q. Protein arginine methyltransferases (PRMTs): Orchestrators of cancer pathogenesis, immunotherapy dynamics, and drug resistance. Biochem Pharmacol. (2024) 221:116048. doi: 10.1016/j.bcp.2024.116048, PMID: 38346542

[14] Sanderson SM Mikhael PG Ramesh V Dai Z Locasale JW. Nutrient availability shapes methionine metabolism in p16/MTAP-deleted cells. Sci Adv. (2019) 5:eaav7769. doi: 10.1126/sciadv.aav7769, PMID: 31249865 PMC6594760

[15] Hu Q Qin Y Ji S Shi X Dai W Fan G. MTAP deficiency-induced metabolic reprogramming creates a vulnerability to cotargeting *de novo* purine synthesis and glycolysis in pancreatic cancer. Cancer Res. (2021) 81:4964–80. doi: 10.1158/0008-5472.CAN-20-0414, PMID: 34385182

[16] Tang B Testa JR Kruger WD. Increasing the therapeutic index of 5-fluorouracil and 6-thioguanine by targeting loss of MTAP in tumor cells. Cancer Biol Ther. (2012) 13:1082–90. doi: 10.4161/cbt.21115, PMID: 22825330 PMC3461815

[17] Kryukov GV Wilson FH Ruth JR Paulk J Tsherniak A Marlow SE. MTAP deletion confers enhanced dependency on the PRMT5 arginine methyltransferase in cancer cells. Science. (2016) 351:1214–8. doi: 10.1126/science.aad5214, PMID: 26912360 PMC4997612

[18] Marjon K Cameron MJ Quang P Clasquin MF Mandley E Kunii K. MTAP deletions in cancer create vulnerability to targeting of the MAT2A/PRMT5/RIOK1 axis. Cell Rep. (2016) 15:574–87. doi: 10.1016/j.celrep.2016.03.043, PMID: 27068473

[19] Mavrakis KJ McDonald ER Schlabach MR Billy E Hoffman GR deWeck A. Disordered methionine metabolism in MTAP/CDKN2A-deleted cancers leads to dependence on PRMT5. Science. (2016) 351:1208–13. doi: 10.1126/science.aad5944, PMID: 26912361

[20] Kalev P Hyer ML Gross S Konteatis Z Chen CC Fletcher M. MAT2A inhibition blocks the growth of MTAP-deleted cancer cells by reducing PRMT5-dependent mRNA splicing and inducing DNA damage. Cancer Cell. (2021) 39:209–224.e211. doi: 10.1016/j.ccell.2020.12.010, PMID: 33450196

[21] Alhalabi O Chen J Zhang Y Lu Y Wang Q Ramachandran S. MTAP deficiency creates an exploit able target for antifolate therapy in 9p21-loss cancers. Nat Commun. (2022) 13:1797. doi: 10.1038/s41467-022-29397-z, PMID: 35379845 PMC8980015

[22] Mu H Zhang Q Zuo D Wang J Tao Y Li Z. Methionine intervention induces PD-L1 expression to enhance the immune checkpoint therapy response in MTAP-deleted osteosarcoma. Cell Rep Med. (2025) 6:101977. doi: 10.1016/j.xcrm.2025.101977, PMID: 39983717 PMC11970323

[23] Reschke R Yu J Flood B Higgs EF Hatogai K Gajewski TF. Immune cell and tumor cell-derived CXCL10 is indicative of immunotherapy response in metastatic melanoma. J Immunother Cancer. (2021) 9:e003521. doi: 10.1136/jitc-2021-003521, PMID: 34593622 PMC8487215

[24] Tokunaga R Zhang W Naseem M Puccini A Berger MD Soni S. CXCL9, CXCL10, CXCL11/CXCR3 axis for immune activation - A target for novel cancer therapy. Cancer Treat Rev. (2018) 63:40–7. doi: 10.1016/j.ctrv.2017.11.007, PMID: 29207310 PMC5801162

[25] Consortium, I.T.P.-C.A.O.W.G. Pan-cancer analysis of whole genomes. Nature. (2020) 578:82–93. doi: 10.1038/s41586-020-1969-6, PMID: 32025007 PMC7025898

[26] Gross MI Demo SD Dennison JB Chen L Chernov-Rogan T Goyal B. Antitumor activity of the glutaminase inhibitor CB-839 in triple-negative breast cancer. Mol Cancer Ther. (2014) 13:890–901. doi: 10.1158/1535-7163.MCT-13-0870, PMID: 24523301

[27] Engstrom LD Aranda R Waters L Moya K Bowcut V Vegar L. MRTX1719 is an MTA-cooperative PRMT5 inhibitor that exhibits synthetic lethality in preclinical models and patients with MTAP-deleted cancer. Cancer Discov. (2023) 13:2412–31. doi: 10.1158/2159-8290.CD-23-0669, PMID: 37552839 PMC10618744

[28] Belmontes B Slemmons KK Su C Liu S Policheni AN Moriguchi J. AMG 193, a clinical stage MTA-cooperative PRMT5 inhibitor, drives antitumor activity preclinically and in patients with MTAP-deleted cancers. Cancer Discov. (2025) 15:139–61. doi: 10.1158/2159-8290.CD-24-0887, PMID: 39282709 PMC11726016

[29] Briggs KJ Cottrell KM Tonini MR Tsai A Zhang M Whittington DA. TNG908 is a brain-penetrant, MTA-cooperative PRMT5 inhibitor developed for the treatment of MTAP-deleted cancers. Transl Oncol. (2025) 52:102264. doi: 10.1016/j.tranon.2024.102264, PMID: 39756156 PMC11832951

[30] Harding JJ Telli M Munster P Voss MH Infante JR DeMichele A. A phase I dose-escalation and expansion study of telaglenastat in patients with advanced or metastatic solid tumors. Clin Cancer Res. (2021) 27:4994–5003. doi: 10.1158/1078-0432.CCR-21-1204, PMID: 34285061 PMC9401498

[31] Tannir NM Agarwal N Porta C Lawrence NJ Motzer R McGregor B. Efficacy and safety of telaglenastat plus cabozantinib vs placebo plus cabozantinib in patients with advanced renal cell carcinoma: the CANTATA randomized clinical trial. JAMA Oncol. (2022) 8:1411–8. doi: 10.1001/jamaoncol.2022.3511, PMID: 36048457 PMC9437824

[32] Ciombor KK Bae SW Whisenant JG Ayers GD Sheng Q Peterson TE. Results of the phase I/II study and preliminary B-cell gene signature of combined inhibition of glutamine metabolism and EGFR in colorectal cancer. Clin Cancer Res. (2025) 31:1437–48. doi: 10.1158/1078-0432.CCR-24-3133, PMID: 39927885 PMC11996605

[33] Gouda MA Voss MH Tawbi H Gordon M Tykodi SS Lam ET. A phase I/II study of the safety and efficacy of telaglenastat (CB-839) in combination with nivolumab in patients with metastatic melanoma, renal cell carcinoma, and non-small-cell lung cancer. ESMO Open. (2025) 10:104536. doi: 10.1016/j.esmoop.2025.104536, PMID: 40359708 PMC12141888

[34] Wang Z Liu F Fan N Zhou C Li D Macvicar T. Targeting glutaminolysis: new perspectives to understand cancer development and novel strategies for potential target therapies. Front Oncol. (2020) 10:589508. doi: 10.3389/fonc.2020.589508, PMID: 33194749 PMC7649373

[35] Varghese S Pramanik S Williams LJ Hodges HR Hudgens CW Fischer GM. The glutaminase inhibitor CB-839 (Telaglenastat) enhances the antimelanoma activity of T-cell-mediated immunotherapies. Mol Cancer Ther. (2021) 20:500–11. doi: 10.1158/1535-7163.MCT-20-0430, PMID: 33361272 PMC7933078

[36] Lobel GP Jiang Y Simon MC. Tumor microenvironmental nutrients, cellular responses, and cancer. Cell Chem Biol. (2023) 30:1015–32. doi: 10.1016/j.chembiol.2023.08.011, PMID: 37703882 PMC10528750

[37] Yoo HC Yu YC Sung Y Han JM. Glutamine reliance in cell metabolism. Exp Mol Med. (2020) 52:1496–516. doi: 10.1038/s12276-020-00504-8, PMID: 32943735 PMC8080614

[38] Schulte ML Fu A Zhao P Li J Geng L Smith ST. Pharmacological blockade of ASCT2-dependent glutamine transport leads to antitumor efficacy in preclinical models. Nat Med. (2018) 24:194–202. doi: 10.1038/nm.4464, PMID: 29334372 PMC5803339

[39] Leone RD Zhao L Englert JM Sun IM Oh MH Sun IH. Glutamine blockade induces divergent metabolic programs to overcome tumor immune evasion. Science. (2019) 366:1013–21. doi: 10.1126/science.aav2588, PMID: 31699883 PMC7023461

[40] Yuan Z Yu T Wang X Meng K Wang T Wang B. Glutamine deprivation confers immunotherapy resistance by inhibiting IFN-gamma signaling in cancer cells. Pharmacol Res. (2025) 213:107643. doi: 10.1016/j.phrs.2025.107643, PMID: 39909124

[41] Yan W Qiu L Yang M Xu A Ma M Yuan Q. CXCL10 mediates CD8(+) T cells to facilitate vessel normalization and improve the efficacy of cetuximab combined with PD-1 checkpoint inhibitors in colorectal cancer. Cancer Lett. (2023) 567:216263. doi: 10.1016/j.canlet.2023.216263, PMID: 37354983

[42] Cheng CC Chang J Ho AS Sie ZL Peng CL Wang CL. Tumor-intrinsic IFNalpha and CXCL10 are critical for immunotherapeutic efficacy by recruiting and activating T lymphocytes in tumor microenvironment. Cancer Immunol Immunother. (2024) 73:175. doi: 10.1007/s00262-024-03761-y, PMID: 38953994 PMC11219622

[43] Yin T Mou S Zhang H Dong Y Yan B Huang W. CXCL10 could be a prognostic and immunological biomarker in bladder cancer. Discov Oncol. (2024) 15:148. doi: 10.1007/s12672-024-00982-6, PMID: 38720149 PMC11078901

[44] Burke SJ Goff MR Lu D Proud D Karlstad MD Collier JJ. Synergistic expression of the CXCL10 gene in response to IL-1beta and IFN-gamma involves NF-kappaB, phosphorylation of STAT1 at Tyr701, and acetylation of histones H3 and H4. J Immunol. (2013) 191:323–36. doi: 10.4049/jimmunol.1300344, PMID: 23740952

[45] Decout A Katz JD Venkatraman S Ablasser A. The cGAS-STING pathway as a therapeutic target in inflammatory diseases. Nat Rev Immunol. (2021) 21:548–69. doi: 10.1038/s41577-021-00524-z, PMID: 33833439 PMC8029610

[46] Mowen KA Tang J Zhu W Schurter BT Shuai K Herschman HR. Arginine methylation of STAT1 modulates IFNalpha/beta-induced transcription. Cell. (2001) 104:731–41. doi: 10.1016/s0092-8674(01)00269-0, PMID: 11257227

[47] Harris DP Bandyopadhyay S Maxwell TJ Willard B DiCorleto PE. Tumor necrosis factor (TNF)-alpha induction of CXCL10 in endothelial cells requires protein arginine methyltransferase 5 (PRMT5)-mediated nuclear factor (NF)-kappaB p65 methylation. J Biol Chem. (2014) 289:15328–39. doi: 10.1074/jbc.M114.547349, PMID: 24753255 PMC4140890

[48] Kim H Kim H Feng Y Li Y Tamiya H Tocci S. PRMT5 control of cGAS/STING and NLRC5 pathways defines melanoma response to antitumor immunity. Sci Transl Med. (2020) 12:eaaz5683. doi: 10.1126/scitranslmed.aaz5683, PMID: 32641491 PMC7508354

[49] Limagne E Nuttin L Thibaudin M Jacquin E Aucagne R Bon M. MEK inhibition overcomes chemoimmunotherapy resistance by inducing CXCL10 in cancer cells. Cancer Cell. (2022) 40:136–152 e112. doi: 10.1016/j.ccell.2021.12.009, PMID: 35051357

[50] Lim RJ Salehi-Rad R Tran LM Oh MS Dumitras C Crosson WP. CXCL9/10-engineered dendritic cells promote T cell activation and enhance immune checkpoint blockade for lung cancer. Cell Rep Med. (2024) 5:101479. doi: 10.1016/j.xcrm.2024.101479, PMID: 38518770 PMC11031384

